# Late Presentation of Galloway-Mowat Syndrome (GAMOS) Associated With Membranous Nephropathy: A Case Report

**DOI:** 10.7759/cureus.82108

**Published:** 2025-04-11

**Authors:** Eugene K Yeboah, Steven Salvatore, Sandeep Sasidharan, Sulayman Khan, Subodh Saggi

**Affiliations:** 1 Department of Internal Medicine, State University of New York Downstate Medical Center, Brooklyn, USA; 2 Department of Pathology and Laboratory Medicine, Weill Cornell Medicine, New York, USA; 3 Department of Nephrology, State University of New York Downstate Medical Center, Brooklyn, USA

**Keywords:** galloway-mowat syndrome, gamos, gamos with membranous nephropathy, steroid-resistant nephrotic syndrome, wdr73 gene

## Abstract

We present a rare case of Galloway-Mowat syndrome (GAMOS) in an elderly patient with a WD repeat domain 73 (WDR73) gene deletion. A 64-year-old man with recurrent deep vein thrombosis on anticoagulation, intermittent atrial fibrillation, ulcerative colitis, apical hypertrophic cardiomyopathy, hypertension, and biopsy-proven membranous nephropathy (MN) presented with urinary frequency associated with frothing. His physical examination was unremarkable. His workup revealed worsening proteinuria, which was previously controlled with tacrolimus, low-dose steroid, and enalapril. His laboratory workup showed a serum creatinine level of 1 mg/dL, albumin at 3.5 g/dL, a urine-protein-to-creatinine ratio of 2,800 mg/g, complement 3 (C3) at 127 mg/dL, and complement 4 at 41 mg/dL. Although APOL1 testing was negative, a pathologic deletion in the WDR73 gene was identified. Repeat kidney biopsy showed MN with rare global glomerulosclerosis with mild interstitial fibrosis. Immunofluorescence showed a granular pattern along capillary walls for immunoglobulin G (IgG), C3, kappa, and lambda light chains. However, it was negative for M-type phospholipase A2 receptor (PLA2R), thrombospondin type 1 domain-containing 7A, and neural epidermal growth factor-like 1. An electron microscope showed a markedly irregular contour of the basement membrane with subepithelial and intramembranous electron-dense deposits. Our patient was diagnosed with GAMOS associated with MN, and intensive management of proteinuria was initiated. We present this unique patient with GAMOS and PLA2R-negative MN in an adult.

## Introduction

Galloway-Mowat syndrome (GAMOS) is an uncommon autosomal recessive disorder characterized by various neurological and renal abnormalities [1]. Renal manifestations range from asymptomatic proteinuria to early-onset steroid-resistant nephrotic syndrome. It was originally described in 1968 by Galloway and Mowat in two siblings with the triad of congenital nephrotic syndrome, microcephaly, and hiatus hernia [1]. The majority of patients usually die within a few years of the onset of the disease, and the commonest causes of death are nephrotic syndrome or seizures. Steroid-resistant nephrotic syndrome, a common renal manifestation in GAMOS, is associated with poor prognosis and often progresses to end-stage renal disease [1]. The estimated prevalence is less than 1/1,000,000 [2]. Homozygous mutations in the WD repeat domain 73 (WDR73) were first implicated in patients with GAMOS in 2014 [3]. We report a rare case of an adult who presented with only proteinuria and was found to have GAMOS with a deletion of the WDR73 gene.

## Case presentation

A 64-year-old man with a history of recurrent deep vein thrombosis on anticoagulation, intermittent atrial fibrillation, ulcerative colitis, apical hypertrophic cardiomyopathy, hypertension, and biopsy-proven membranous nephropathy (MN) presented with worsening urinary frequency associated with frothing despite being compliant with all his medications for his previously diagnosed MN. The patient’s initial home medications included sulfasalazine 1 g three times daily, folic acid 1 mg daily, apixaban 2.5 mg twice daily, rosuvastatin 20 mg daily, famotidine 20 mg daily, tacrolimus 5 mg twice daily, prednisolone 5 mg daily, metoprolol succinate 25 mg daily, and enalapril 5 mg daily. The patient’s initial kidney biopsy, which confirmed MN, was done in Barbados. The patient denied smoking, alcohol, or illicit drug use but had a significant family history of cancer. The patient’s mother and sister both had breast cancer, and the mother also had colon cancer. He had inguinal hernia repair at 5 and 63 years, respectively. He also had a lipoma removed.

The patient's initial physical examination and vital signs were temperature 97.5°F, heart rate 79 bpm, respiratory rate 16/minute, saturation on room air 100%, blood pressure 132/66 mmHg, and BMI 25.9 kg/m^2^. The patient was euvolemic, not jaundiced, and not pale, and had no pedal edema. The patient’s chest was clinically clear, and abdominal examination was unremarkable. He was conscious, alert, and oriented in terms of time, place, and person. Table 1 summarizes the patient’s extensive laboratory workup, which was largely within normal limits except for elevated proteinuria with a urine-protein-to-creatinine ratio of 2,800 mg/g.

**Table 1:** Patient’s laboratory workup IU: international units; hpf: high-power field; lpf: low-power field

Parameter	Patient values	Reference range
Comprehensive metabolic panel		
Sodium	138 mmol/L	136-145 mmol/L
Potassium	3.9 mmol/L	3.5-5.1 mmol/L
Calcium	8.7 mg/dL	8.2-10 mg/dL
Chloride	103 mmol/L	98-107 mmol/L
Magnesium	1.6 mg/dL	1.9-2.7 mg/dL
Creatinine	1.0 mg/dL	0.7-1.3 mg/dL
Blood urea nitrogen	12 mg/dL	7-25 mg/dL
Carbon dioxide	28 mmol/L	21-31 mmol/L
Glucose	93 mg/dL	70-99 mg/dL
Anion gap	11 mmol/L	10-20 mmol/L
Estimated glomerular filtration rate	82 mL/minute/1.73 m²	>60 mL/minute/1.73 m²
Liver function test		
Total bilirubin	0.6 mg/dL	0.3-1 mg/dL
Albumin	3.5 g/dL	3.5-5.7 g/dL
Total protein	5.8 g/dL	6-8.3 g/dL
Aspartate aminotransferase	21 U/L	13-39 U/L
Alanine aminotransferase	13 U/L	7-52 U/L
Alkaline phosphatase	82 U/L	34-104 U/L
Glycated hemoglobin (HbA1C)	4.6%	<5.7%
Complete blood count		
Hemoglobin	14.6 g/dL	14-18 g/dL
White blood count	8.41k/μL	3.5-10.8k/μL
Platelet	152k/μL	130-400k/μL
Hematocrit	43.2%	42%-52%
Urinalysis		
Appearance	Slightly cloudy	Clear
pH	5	5-8
Specific gravity	1.024	1.005-1.030
Urine glucose	Negative	Negative
Urine blood	Small	Negative
Urine creatinine	282 mg/dL	20-320 mg/dL
Urine protein	>500 mg/dL	Negative
Urine nitrite	Negative	Negative
Leucocyte esterase	Negative	Negative
White blood cells (urine)	<1/hpf	0-5/hpf
Urine cast	21/lpf	0-2/lpf
Urine protein-creatinine ratio	2,800 mg/g	<150 mg/g
Coagulation		
Prothrombin time	12.9 seconds	10.8-13.7 seconds
Activated partial thromboplastin time	31.3 seconds	25-35 seconds
International normalized ratio	1.1	<1
Glomerulopathy workup		
Complement (C3) levels	127 mg/dL	86-184 mg/dL
Complement (C4) levels	41 mg/dL	20-58 mg/dL
Complement total (CH50)	57	42-95 U/mL
Infectious workup		
Human immunodeficiency virus 1/2 antigen/antibodies	Negative	Negative
Hepatitis C	Nonreactive	Nonreactive
Hepatitis B surface antigen	Nonreactive	Nonreactive
Tuberculosis QuantiFERON gold	Negative	Negative
Autoimmune workup		
Antinuclear antibody	Negative	Negative
Anti-double-stranded DNA	<1 IU/mL	<29 IU/mL
Genetic test		
Apolipoprotein L1	Negative	Negative
WDR73 gene deletion	Positive	Negative

Imaging

**Figure 1:**
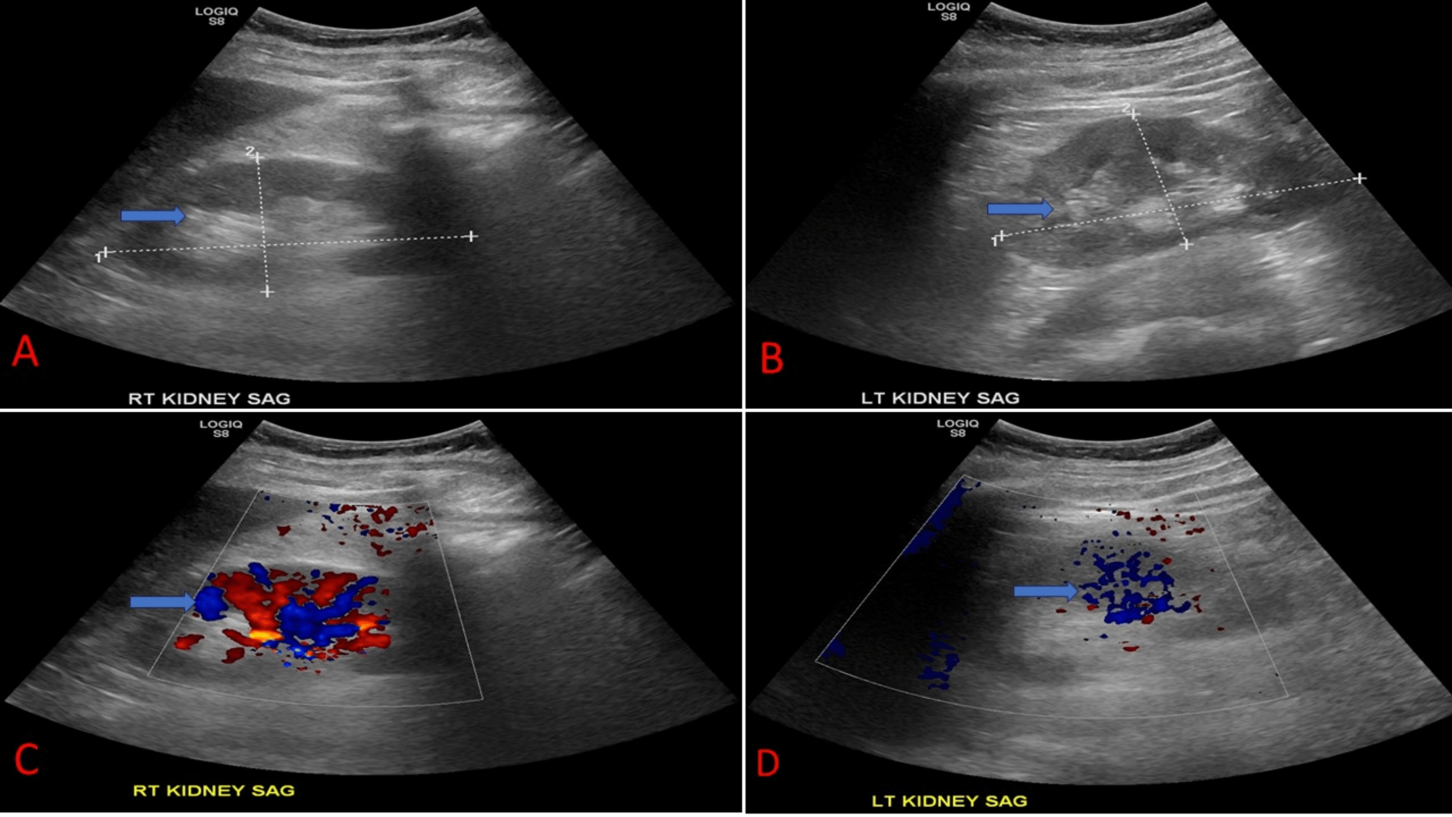
Kidney ultrasound of our patient.(A) Right kidney of size 10.6 x 5.3 x 5.0 cm with increased corticomedullary differentiation (blue arrow). (B) Left kidney of size 10.6 x 5.3 x 4.9 cm with increased corticomedullary differentiation (blue arrow). (C) Doppler images showing blood flow (blue arrow) of the right kidney. (D) Doppler images showing blood flow (blue arrow) of the left kidney

The kidney ultrasound is represented in Figure 1. It showed increased echogenicity but normal parenchyma thickness and contour, and no pelvicalyceal dilatation, calculi, cysts, or solid masses in both kidneys. The increased echogenicity and absence of cysts/masses suggest chronic nephropathy.

Kidney biopsy

**Figure 2:**
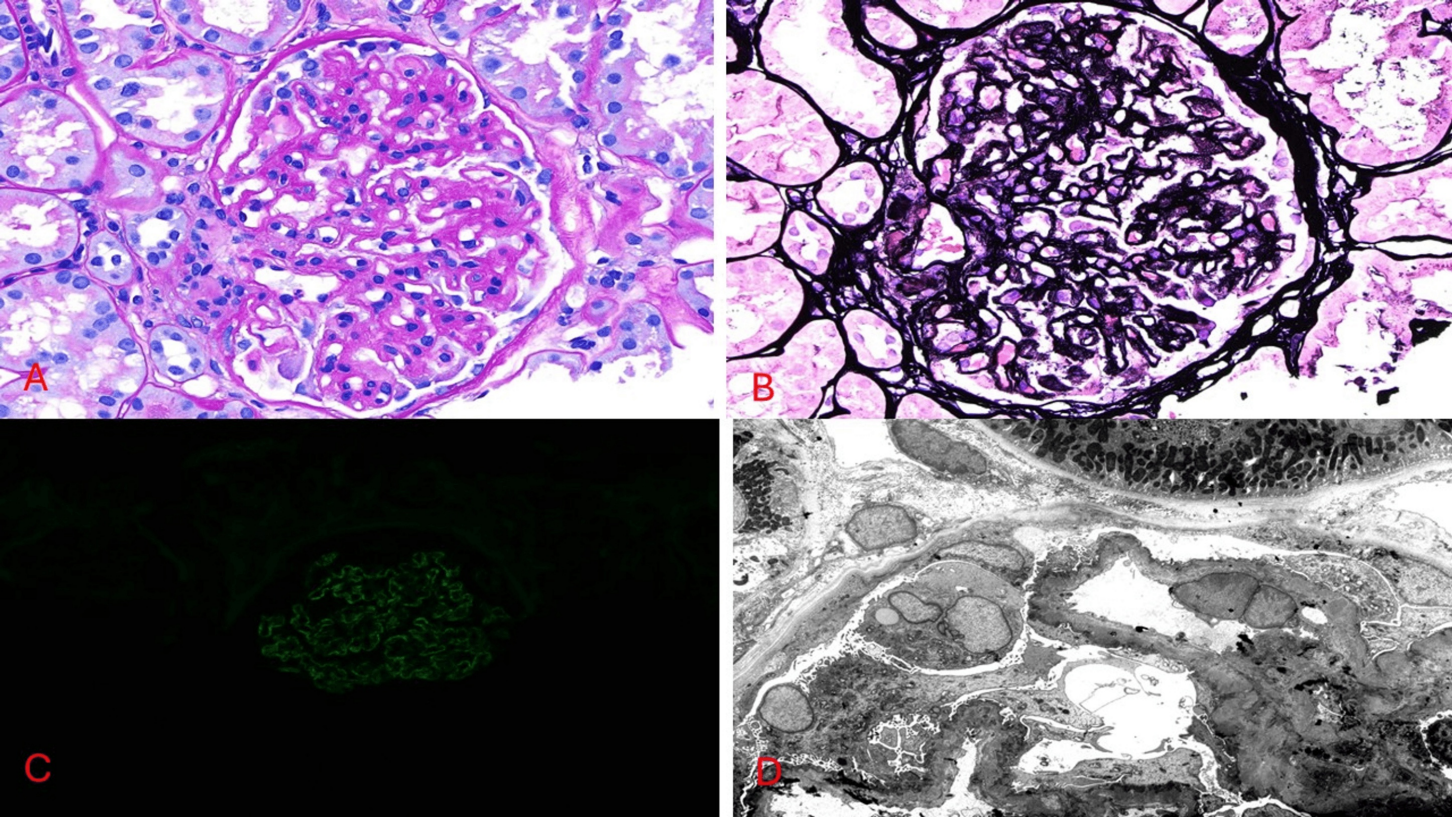
The patient's biopsy images. (A) Glomerulus showing irregular capillary wall thickening, PAS stain (40×). (B) Glomerulus revealing capillary spikes by Jones silver staining (40×). (C) Granular capillary wall staining for IgG on immunofluorescence (IgG, 40×). (D) Glomerular capillary loops with subepithelial and intramembranous electron-dense deposits and overlying complete foot process effacement (electron microscopy, 2,500×) PAS: periodic acid-Schiff; IgG: immunoglobulin G

A repeat kidney biopsy was done to work up the worsening proteinuria. The results of the repeat kidney biopsy are presented in Figure 2. The biopsy consisted of three cores of renal cortex and medulla with 29 glomeruli. Five glomeruli were globally sclerosed. The remaining glomeruli showed irregular capillary wall thickening and spike formation on Jones silver staining, indicative of MN. Deposits were confirmed by immunofluorescence and were positive for IgG and complement 3. Immunofluorescence staining was negative for phospholipase A2 receptor (PLA2R), thrombospondin type 1 domain-containing 7A (THSD7A), and neural epidermal growth factor-like 1 (NELL-1). Electron microscopy revealed subepithelial and intramembranous electron-dense deposits with reactive basement membrane spikes and complete foot process effacement. No mesangial or subendothelial deposits were present.

The clinical findings of adult-onset proteinuria (urine protein-to-creatinine ratio of 2,800 mg/g), together with kidney biopsy-confirmed MN but negative PLA2R/THSD7A/NELL-1, support the diagnosis of GAMOS-associated MN in the context of WADR73 gene deletion. The patient was started on cyclosporin by a nephrologist in Barbados. We recommended rituximab infusion to decrease the risk of progression of kidney disease, as patients had a poor response to calcineurin inhibitors.

## Discussion

We report a rare case of an adult who presented with only proteinuria and was found to have GAMOS with the deletion of the WDR73 gene. GAMOS was originally described in 1968 [1]. Since then, about 60 cases of GAMOS have been reported [4]. The clinical spectrum of GAMOS is vast, and it includes craniofacial dysmorphism, developmental delay, seizure disorder, hypotonia, psychomotor retardation, extremities abnormalities, and diverse renal manifestations [4]. Hiatus hernia was initially part of the diagnostic criteria. However, it is no longer considered a core clinical feature of GAMOS [5]. Our case is a unique form of GAMOS because our patient presented in adulthood and without any neurological symptoms, which is almost always required to make a diagnosis.

Renal manifestations of GAMOS vary from isolated proteinuria to steroid-resistant nephrotic syndrome [3,6,7]. There were no renal abnormalities in a few reported cases [3,6,7]. Clinical renal variability of GAMOS exists even within the same family. For example, two siblings carrying the same pathogenic mutation of WDR73 developed contrasting renal pathologies [3]. One sibling developed nephrotic syndrome at five years, which rapidly led to chronic renal failure and death after a month of diagnosis [7]. The other sibling who had the same pathologic variant of WDR73 had no renal disease even at seven years [3]. Our patient had a pathologic variant of WDR73 but presented with an isolated proteinuria, which also lies in the spectrum of renal disease in GAMOS.

There is genetic heterogeneity of GAMOS. Loss-of-function mutation of the WDR73 gene has been reported in different families with GAMOS [3,6]. Recessive variants in the genes encoding for kinases, endopeptidases, and other small-sized protein complexes, such as 0-sialoglycoprotein endopeptidase (OSGEP), TP53-regulating kinase (TP53RK), TP53RK binding protein, and L-antigen family member 3, have also been identified and linked to GAMOS in different families [7]. A recent in vivo knockdown study of OSGEP and TP53RK led to pathologies in the actin cytoskeleton as well as a reduction in the human podocyte migration rate [7]. These pathological manifestations led to the development of nephrotic syndrome [7]. Our patient had a gene deletion of WDR73, which possibly led to the worsening proteinuria. A case report involving four families showed findings consistent with GAMOS; the affected individuals had a mutation in the Nucleoporin 133 gene [8]. Another case report of four families identified mutations in the gene encoding for the zinc-finger domain of the transcriptional regulator PR/SET domain 15 that resulted in nephrotic syndrome within a few months of life and other features of GAMOS [9]. Our patient's family history was negative for proteinuria or kidney disease.

GAMOS is a rare disease entity with no existing management guidelines. Genetic testing helps in making a diagnosis, but currently, there are no treatment modalities. Management includes managing the comorbidities associated with it. In our patient, an effort was made to reduce proteinuria with medications to reduce the progression of kidney disease. In cases where the disease is detected in childhood, early referrals for genetic counseling and testing, as well as management of comorbidities, are primarily the treatment modalities.

## Conclusions

Our case highlights the late onset of GAMOS with nephropathy but without neurologic manifestations. This form of MN with an unknown responsible antigen, with negative staining for PLA2R, THSD7A, and NELL-1, is potentially related to the detected underlying deletion of the WDR73 gene as an atypical presentation of GAMOS. Genetic testing is key in clenching the diagnosis in suspected cases.

## References

[1] Galloway WH, Mowat AP (1968). Congenital microcephaly with hiatus hernia and nephrotic syndrome in two sibs. J Med Genet.

[2] Domingo-Gallego A, Furlano M, Pybus M (2019). Novel homozygous OSGEP gene pathogenic variants in two unrelated patients with Galloway-Mowat syndrome: case report and review of the literature. BMC Nephrol.

[3] Colin E, Huynh Cong E, Mollet G (2014). Loss-of-function mutations in WDR73 are responsible for microcephaly and steroid-resistant nephrotic syndrome: Galloway-Mowat syndrome. Am J Hum Genet.

[4] Lin PY, Tseng MH, Zenker M (2018). Galloway-Mowat syndrome in Taiwan: OSGEP mutation and unique clinical phenotype. Orphanet J Rare Dis.

[5] Cooperstone BG, Friedman A, Kaplan BS (1993). Galloway-Mowat syndrome of abnormal gyral patterns and glomerulopathy. Am J Med Genet.

[6] Rosti RO, Dikoglu E, Zaki MS (2016). Extending the mutation spectrum for Galloway-Mowat syndrome to include homozygous missense mutations in the WDR73 gene. Am J Med Genet A.

[7] Braun DA, Rao J, Mollet G (2017). Mutations in KEOPS-complex genes cause nephrotic syndrome with primary microcephaly. Nat Genet.

[8] (2019). Correction. Ann Neurol.

[9] Mann N, Mzoughi S, Schneider R (2021). Mutations in PRDM15 are a novel cause of Galloway-Mowat syndrome. J Am Soc Nephrol.

